# The Naked Mole Rat: A Unique Example of Positive Oxidative Stress

**DOI:** 10.1155/2019/4502819

**Published:** 2019-02-07

**Authors:** Frédéric Saldmann, Melanie Viltard, Christine Leroy, Gérard Friedlander

**Affiliations:** ^1^Fondation pour la Recherche en Physiologie, Brussels, Belgium; ^2^Service de Physiologie et Explorations Fonctionnelles, Hôpital Européen Georges Pompidou, Assistance Publique-Hôpitaux de Paris, Paris, France; ^3^Université Paris Descartes, Faculté de Médecine, Paris, France; ^4^INSERM UMR_S1151 CNRS UMR8253 Institut Necker-Enfants Malades (INEM), Paris, France

## Abstract

The oxidative stress theory of aging, linking reactive oxygen species (ROS) to aging, has been accepted for more than 60 years, and numerous studies have associated ROS with various age-related diseases. A more precise version of the theory specifies that mitochondrial oxidative stress is a direct cause of aging. The naked mole rat, a unique animal with exceptional longevity (32 years in captivity), appears to be an ideal model to study successful aging and the role of ROS in this process. Several studies in the naked mole rat have shown that these animals exhibit a remarkable resistance to oxidative stress. At low concentrations, ROS serve as second messengers, and these important intracellular signalling functions are crucial for the regulation of cellular processes. In this review, we examine the literature on ROS and their functions as signal transducers. We focus specifically on the longest-lived rodent, the naked mole rat, which is a perfect example of the paradox of living an exceptionally long life with slow aging despite high levels of oxidative damage from a young age.

## 1. Introduction

Aging is defined as a progressive decline in physiological function that ultimately leads to decreased reproductive rate and increased mortality. More than 60 years ago, Harman first proposed that aging could be attributed to the deleterious effects of free radicals produced as natural by-products of aerobic metabolism [1]. The free radical theory of aging (FRTA) is based on the hypothesis that dysfunctions observed during aging and a range of age-associated pathologies are due to the accumulation of oxidative damage to biological macromolecules (e.g., DNA damage, lipid peroxidation, and nonrepairable protein oxidation) by reactive oxygen and nitrogen species [1, 2].

A more precise version of the free radical theory of aging, called the mitochondrial free radical theory of aging (MFRTA), specifies that mitochondria are the main sources of ROS generation and are also the targets of deleterious effects: oxidative damages to mitochondrial DNA, mitochondrial proteins, or phospholipids are assumed to directly cause aging [3–6].

Data supporting the FRTA and the MFRTA theories come from many studies that have shown that the production of ROS and oxidative damage accumulate with age [7–10]. More importantly, human healthy centenarians (>100 years) have lower levels of oxidative stress compared to younger controls (70–99 years) [11, 12]. In addition, excessive ROS production is associated with many age-related pathologies such as diabetes, neurodegenerative diseases, and cancer [13–16]. Multiple comparative studies have shown that the rates of mitochondrial ROS (mtROS) production and oxidative damage to mitochondrial DNA are negatively correlated with maximal lifespan and that many long-lived animals have lower rates of production than short-lived animals [17–23]. In agreement with the MFRTA, several studies showed that some nutritional interventions (caloric, protein, or methionine restriction but not glucose or lipid restriction) that increase longevity in rodents also decrease the rate of mitochondrial ROS production in several different tissues [24–29].

## 2. The Naked Mole Rat: An Exception to the Rule

Naked mole rats (*Heterocephalus glaber*), first described by Rüppell in 1842, are the longest living rodents known. About the size of a mouse, this hairless poikilothermic eusocial rodent lives almost ten times as long as a mouse, with a maximum lifespan of about 32 years in captivity and 17 years in the wild [30, 31]. The naked mole rat is an outlier on the allometric relationship between body mass and maximum lifespan [32–34]. In addition to its extreme longevity, naked mole rats exhibit other exceptional characteristics including high fertility even in queens of 30 years of age, negligible senescence, and resistance to cancer, diabetes, neurodegenerative diseases, and many other age-related pathologies [35, 36]. Hence, the naked mole rat appears to be a perfect animal model to study healthy aging and the role of free radicals in this process.

Several studies have investigated the production of free radicals and oxidative damages in the naked mole rat, and the results are puzzling. Despite remarkably long lives, some tissues of the naked mole rat, such as arteries, produce higher amounts of ROS (from cytoplasmic and mitochondrial sources) as compared to these tissues from the short-lived mouse [37]. Importantly, the arteries of naked mole rats are highly resistant to the proapoptotic effects of ROS *in vitro*, whereas those of the mouse are not [37]. In addition, it has been shown that the rate of mtROS production in the naked mole rat heart is statistically similar to that of the mouse [21, 38]. However, mtROS production in the naked mole rat kidney and skeletal muscle is significantly lower than that in these organs in mice suggesting that mtROS production could be tissue specific or may have tissue-specific functions [38]. It would be interesting to measure mtROS in other vital tissues such as the brain, liver, and lungs in the naked mole rat.

Furthermore, several studies have shown that naked mole rats have high levels of oxidative damages to macromolecules from a young age. Indeed, high levels of oxidative modifications to lipids (urinary isoprostanes, liver malondialdehyde [39, 40]), proteins (carbonyl formation and oxidation of cysteine residues [41, 42]), and DNA (8-oxo-2′-deoxyguanosine [41]) have been observed in urine and in various tissues of the animal. Interestingly, these levels of damages are maintained over a 20-year period without increase. One hypothesis is that further damages are attenuated by an efficient repair system. A limit of these studies is that only damages to macromolecules were investigated: mitochondrial DNA damage has not been studied in naked mole rat tissues. Hence, further studies using this unique animal model are needed as it would be very informative to compare ROS-producing systems from cellular and mitochondrial sources and oxidative damage in nuclear, cytoplasmic, and mitochondrial targets in long-lived naked mole rat and short-lived rodents.

In young naked mole rat protein, carbonyls and oxidation of cysteine residues are observed at high frequency [41, 42]. One highly oxidized protein in the naked mole rat is triosephosphate isomerase (TPI), an important glycolytic enzyme. It is interesting to note that, despite extremely high levels of damage, activity of TPI is not compromised [41]. Moreover, despite high levels of cysteine oxidation, naked mole rat protein structure, integrity, and functionality are better maintained during aging than those of short-lived mice; naked mole rat proteins resist unfolding and are less prone to ubiquitination, and their cells maintain proteostasis through high proteasome activity throughout life [42–45].

Many, but not all, features of the naked mole rat defy the free radical theories of aging. However, there is a recent extension of the theory, called the membrane pacemaker theory of aging [46], which holds true in the naked mole rat. This theory predicts that membrane fatty acid composition has an influence on lipid peroxidation and consequently may be an important determinant of aging and lifespan. Indeed, a study showed that naked mole rat membranes from different tissues contain more fatty acids resistant to peroxidation than do membranes from mice [47]. Thus, the cellular membrane composition of the naked mole rat could partially explain their exceptional longevity.

Studies attempting to confirm the FRTA have often focused on antioxidant systems, as an obvious hypothesis is that long-lived animals must have a superior antioxidant line of defence, neutralizing ROS before it can have harmful effects. However, many studies of antioxidant activity have shown that the levels of endogenous antioxidants are negatively correlated with lifespan (for review, see [48]). An explanation may be that long-lived animals produce lower levels of ROS and do not need high levels of cellular antioxidants and that short-lived animals produce higher levels of ROS [49, 50]. However, unlike many other long-lived animals, the naked mole rat produces large amounts of ROS from cytoplasmic and mitochondrial sources and incurs oxidative damage to macromolecules, but despite this, the levels of most antioxidants evaluated (CuZnSOD, catalase, thioredoxin, glutathione, and glutathione reductase) are lower in naked mole rat tissues than in mouse tissues. Most surprising is the virtual absence of glutathione peroxidase 1 (Gpx1) in naked mole rat tissues, due to an early stop codon in the *Gpx1* mRNA and associated low levels of selenium as compared to mice [51, 52]. Hence, lower antioxidant defences in the naked mole rat could be responsible for the accumulation of unusually elevated levels of oxidative damage in proteins, lipids, and nuclear DNA.

The “naked mole rat exception” raises the question of whether or not ROS (cytoplasmic and mitochondrial) are responsible for aging. Recently, several groups of researchers have challenged the free radical and mitochondrial free radical theory of aging [53–56]. Several criticisms are made against the FRTA. Apart from the naked mole rat, several other animal models refute the FRTA: the long-lived Ames dwarf mice have high levels of mtROS in the cardiovascular system when compared to normal controls [57]. There is a lack of correlation between mtROS and lifespan in *Drosophila* [58]. The long-lived *Mclk1*-mutant mice exhibit high levels of mitochondrial oxidative stress [59, 60]. In addition, the MFRTA is supported mostly by indirect data such as the negative correlation between mitochondrial ROS production and lifespan. These correlations suggest but do not demonstrate a causality, however [61]. One limit shared by most of the studies supporting the MFRTA is the fact that the mtROS production was assessed in isolated mitochondria, which might not reflect mtROS production *in vivo*. Finally, one question is still open as to the direct role of ROS and ROS-mediated damages in aging or rather in the progression of age-related pathologies, i.e., in healthspan or healthy aging [55].

The naked mole rat is a very intriguing animal. Although very small naked mole rats can survive up to 32 years in captivity and probably longer given that this animal defies the Gompertzian laws of mortality [62], as a species, naked mole rats have lived underground for about 24 million years, and during this time, they have evolved a series of biological characteristics that allow them to tolerate the harsh underground environment: they are extremely resistant to hypoxia and to hypercapnia [63–65], and they are also extremely resistant to oxidative stress. The key to their exceptional longevity might be how they mitigate and cope with oxidative damage.

## 3. Stress-Response Hormesis and Aging: A Fundamental Concept

Hormesis is an adaptive response to a variety of stresses, including oxidative stress, which raises an organism's resistance against higher doses of the same stressing agent [66]. Exposure of the naked mole rat to oxidative stress from a young age could enhance its resistance to oxidative damage and lead to a decrease in stress-related aging processes, explaining in part its exceptional longevity. In other words, naked mole rat longevity could be induced by hormetic effects in association with increased ROS: a phenomenon called mitohormesis.

The toxic effects of ROS are only one aspect of their action in living cells. ROS originating from mitochondrial and nonmitochondrial sources act as physiological signal transducers [67]. ROS is a term covering the different chemical species that are formed upon incomplete reduction of oxygen. ROS include the superoxide anion (O2^∙^−), the hydroxyl radical (HO^∙^), and hydrogen peroxide (H_2_O_2_). O2^∙^− and HO^∙^ are highly toxic and highly unstable and do not pass through cell membranes and likely do not serve as signalling molecules [68, 69]. In contrast, H_2_O_2_ is less toxic, uncharged, and relatively stable and can cross cellular membranes freely [70, 71]. ROS, and particularly H_2_O_2_, have characteristics typical of second messengers: synthesis and removal are tightly controlled, their targets are specific, and their actions can be reversed. These properties and the involvement of H_2_O_2_ in a signal transducer have been reviewed [70–72]. Under physiological conditions, the generation of low levels of ROS appears to control a wide range of cellular processes such as cell proliferation, differentiation, migration, stress adaptation, and apoptosis. H_2_O_2_ modulates the activities of central transcription factors such as NF-*κ*B, AP-1, CREB, NOTCH, Nrf2, HSF1, and FoxO [73–76] and tumour suppressors such as PTEN and p53 [77], resulting in effects on signalling pathways mediated by ERK, MAPK, PI3K/AKT, JNK, and JAK/STAT [78, 79].

In the naked mole rat, vascular endothelial and smooth muscle cells of the carotid artery produce high levels of H_2_O_2_ and are resistant to H_2_O_2_-induced apoptosis [37]. It could be that in the naked mole rat, H_2_O_2_ acts as a critical signalling molecule to promote stress resistance, health, and longevity. In support of this hypothesis, Nrf2 signalling activity is constitutively upregulated in naked mole rat fibroblasts [80] and highly overexpressed (6-fold) in the liver as compared to mice [81, 82]. In addition, naked mole rat fibroblasts also have significantly higher levels of p53 protein (50-fold) and activity (15-fold) [80], and the naked mole rat liver has 2-fold higher *p53* gene expression than observed in the short-lived mouse [83]. Cytoprotective mechanisms induced by p53 and Nrf2 protect cells against proliferation-induced damage, increase degradation of damaged proteins and organelles, and maintain the integrity of both genome and proteome [48]. It is not known whether Nrf2 or p53 expression is induced as a result of oxidative stress in naked mole rat cells, but it would be important to investigate whether ROS, and H_2_O_2_ in particular, mediate signalling in this process.

Another interesting example is the possible modulation of the expression of tumour suppressor gene *PTEN* by ROS [84]. Interestingly, naked mole rats have 17 copies of *PTEN* pseudogenes (*PTENps*) [85]. A recent study proposed that duplications of the *p53* gene (20 copies) in the elephant may help this large animal avoid cancer [86]. Similarly, the multiple copies of *PTENps* in the naked mole rat may contribute to its unusual resistance to cancer. As it is not known if the high expression of ROS influences the expression of *PTEN* or *PTENps*, further studies are needed. In addition, the proteome of naked mole rats exhibits high levels of cysteine [42]. The functional significance of this high cysteine content is unknown, but activation of redox cascades through H_2_O_2_-mediated cysteine oxidation is well-characterized [87] and could explain the extreme longevity of the naked mole rat.

ROS also play many critical roles in the immune system and are deeply involved in various aspects of the immune response such as host defence mechanisms against infection, inflammasome activation, immune cell activation, and immune suppression [88]. Studies of the naked mole rat immune system are limited. A recent study demonstrated that the naked mole rat spleen has significant anatomical and morphological differences compared to mouse spleen with higher abundance of macrophages better equipped for phagocytosis [89]. It is speculated that a higher abundance of macrophages with greater phagocytic capability and greater induction of proinflammatory cytokines may result in a strong immune system. During the phagocytic oxidative burst, ROS, as essential components of the antimicrobial/antiviral repertoire of macrophages, lead to the elimination of exogenous pathogens [90]. Although it is not known whether naked mole rat macrophages produce more ROS than macrophages of mice, the involvement of ROS in macrophage phagocytic capability and cytokine production should be investigated further.

As a poikilothermic mammal, naked mole rats have lower body temperatures (30-33°C) than other small rodents [91]. Lowering the body temperature either by genetic manipulation in mice or by lowering external temperature in rotifers, fishes, and worms extends the lifespan [92–95]. Surprisingly, moderately lowering temperature has been shown to induce mitochondrial O2^∙^− leakage and H_2_O_2_ production [96]. These findings suggest that increased ROS formation, observed in poikilothermic mammals such as the naked mole rat, could promote health and extend the lifespan.

It is clear that the increased mitochondrial metabolism and ROS production have no detrimental impacts on the naked mole rat health and longevity. One hypothesis is that high levels of ROS could have positive effects by inducing adaptive responses that ultimately lead to increased stress resistance and antioxidant defence, which would extend the lifespan (Table 1).

## 4. Conclusions

For more than 60 years, researchers have been studying the deleterious effects of ROS in cells and organisms. Numerous evidences suggest that aging organisms produce ROS at a higher rate and resulting oxidative damages could directly promote aging and/or age-related pathologies.

In terms of physiology, the naked mole rat is an outlier: the naked mole rat can live an exceptionally long life according to its relatively small body mass, and it seems to have a negligible aging process. Surprisingly, the long-lived naked mole rat produces a significant amount of ROS both from cytoplasmic and mitochondrial sources and a high level of oxidative damages to macromolecules. Hence, the naked mole rat represents a great opportunity to study the oxidative stress and its resistance in the context of a healthy successful aging process.

In the wild, naked mole rats live in an extremely harsh environment and are constantly exposed to various stresses: high oxidative damage, temporary hypoxia and hypercapnia, absence of light, and low food availability. Hence, these rodents may have developed adaptive responses to cope with theses stresses. Indeed, increased mitochondrial metabolism and ROS formation in the naked mole rat could induce adaptive responses that ultimately lead to increased stress resistance and extended lifespan relative to other mammals of similar size.

## Figures and Tables

**Table 1:** Potential positive effects of ROS in naked mole rats.

	Type of ROS	Gene/mechanisms	Naked mole rat	Ref.
Cellular homeostasis	H_2_O_2_ [75]	*Nrf2*	Fibroblasts: Nrf2 ↗ Liver: Nrf2 ↗	[80–82]
	H_2_O_2_ [77]	*p53*	Fibroblasts: p53 ↗ Liver: p53 ↗	[80, 83]
	H_2_O_2_ [84]	*PTEN*	17 copies of *PTEN* pseudogenes	[85]

Immune system	O_2_^∙^-/H_2_O_2_ [90]	Oxidative burst	Macrophages ↗	[89]

Thermal regulation	O_2_^∙^-/H_2_O_2_ [96]	Hypothermia	Low body temperature 30-33°C	[91]
